# Source-Dependent Phenotypic Differences in Canine Olfactory Ensheathing Cell Cultures from Olfactory Bulb and Mucosa

**DOI:** 10.3390/biomedicines13123120

**Published:** 2025-12-18

**Authors:** Tomasz Gębarowski, Aldona Głowa, Małgorzata Tarnowska, Dawid Jeżewski, Piotr Kuropka, Radomir Henklewski, Maciej Janeczek, Benita Wiatrak

**Affiliations:** 1 Department of Animal Physiology and Biostructure, Faculty of Veterinary Medicine, Wroclaw University of Environmental and Life Sciences, 50-375 Wroclaw, Poland; tomasz.gebarowski@upwr.edu.pl (T.G.); maciej.janeczek@upwr.edu.pl (M.J.); 2 Department of Pharmacology, Faculty of Medicine, Wroclaw Medical University, J. Mikulicza-Radeckiego 2, 50-345 Wroclaw, Poland; aldona.glowa@umw.edu.pl; 3 Division of Histology and Embryology, Department of Biostructure and Animal Physiology, Wroclaw University of Environmental and Life Sciences, Cypriana K. Norwida 25, 50-375 Wroclaw, Poland; malgorzata.tarnowska@upwr.edu.pl (M.T.); piotr.kuropka@upwr.edu.pl (P.K.); 4 Veterinary Biotechnology Student Science Club “Refectio”, Department of Biostructure and Animal Physiology, Faculty of Veterinary Medicine, Wroclaw University of Environmental and Life Sciences, Kożuchowska 1, 51-631 Wroclaw, Poland; 123527@student.upwr.edu.pl; 5 Department of Veterinary Surgery, Institute of Veterinary Medicine, Faculty of Biological and Veterinary Sciences, Nicolaus Copernicus University in Torun, 87-100 Torun, Poland; radomir.h@gmail.com

**Keywords:** olfactory ensheathing cells (OECs), neuroregeneration, canine model, olfactory mucosa

## Abstract

**Background/Aim**: Olfactory ensheathing cells (OECs) are widely studied for neural repair, yet OB- and OM-derived primary cultures differ in accessibility and cellular composition. This study aimed to establish donor-matched canine OB- and OM-derived primary cultures using harmonized isolation conditions and to quantify source-dependent differences in culture composition and proliferative activity. **Materials and Methods**: Olfactory bulbs (OBs) and olfactory mucosa (OM) were collected post-mortem from client-owned dogs (*n* = 10). Primary cultures were established under identical enzymatic dissociation and culture conditions. Culture composition was quantified by immunocytochemistry using p75^NTR (OEC marker) and fibronectin (fibroblast-associated marker), with an epithelial fraction assessed morphologically in OM. Proliferation was assessed by Ki-67 labeling using the Muse^®^ Ki-67 kit (*n* = 5 donors/group). **Results**: Both tissues yielded viable primary cultures. OB-derived cultures had a higher OEC fraction than OM-derived cultures (60.7 ± 6.4% vs. 39.0 ± 6.2%), whereas OM cultures consistently included an epithelial component (27.0 ± 6.6%). Ki-67 labeling was higher in OB-derived cultures than OM-derived cultures (30.2 ± 6.2% vs. 13.0 ± 2.5%; Welch’s *t*-test *p* = 0.0018). **Conclusions**: Canine OB and OM generate source-distinct primary cultures under standardized conditions: OB-derived cultures are OEC-enriched and more proliferative in vitro, while OM-derived cultures are more heterogeneous. These findings inform future optimization of OM-based protocols and motivate functional assays to test regenerative efficacy.

## 1. Introduction

Spinal cord regeneration remains one of the greatest challenges in contemporary medicine—both veterinary and human. Such injuries result in permanent motor and sensory deficits, and their treatment requires long-term care, intensive rehabilitation, and often advanced surgical interventions. In dogs, this problem is particularly prevalent due to the high incidence of intervertebral disk disease, traffic accidents, and falls [1], whereas in humans, the main causes are traffic accidents, sports injuries, and falls from height [2].

Olfactory ensheathing cells (OECs) are specialized glial cells of the primary olfactory system that support the lifelong regeneration of olfactory receptor neuron axons and their accurate targeting from the olfactory mucosa to the olfactory bulb. This unique biology—combining axon guidance, trophic support, debris clearance, and adaptability to distinct tissue environments—has made OECs a widely studied cell type in the context of neural repair and regenerative strategies within the nervous system. In dogs, which represent a clinically relevant large-animal species, establishing reproducible, donor-informed methods for isolating and characterizing OEC-containing cultures from accessible tissue sources remains important for downstream experimental and translational work.

Numerous in vivo and in vitro studies over the past decades have deepened our understanding of the biological mechanisms governing nervous system repair after injury [3,4], and the development of biomaterials supporting peripheral nerve regeneration has opened new therapeutic avenues [5]. Among cellular strategies, special attention has been given to OECs—specialized glial cells that, under natural conditions, enable the regeneration of olfactory neuron axons throughout life [5]. These cells can be obtained from both the olfactory bulb and the nasal mucosa [6]. OECs promote axonal regrowth, guide regenerating fibers, and secrete neurotrophic factors, making them a promising therapeutic tool for spinal cord injuries in both dogs and humans [7,8,9]. Great expectations are also associated with combining OECs with other strategies, such as mesenchymal stem cells (MSCs) or collagen carriers, to enhance survival and integration of transplanted cells at the injury site [6,10]. In this study, because proliferative capacity may differ between sources, we quantified cell-cycle activity using Ki-67, a nuclear marker present in G1/S/G2/M but absent in G0, and report the proliferative fraction as the Ki-67 labeling index.

Although olfactory ensheathing cells (OECs) derived from the olfactory bulb (OB) and olfactory mucosa (OM) have been compared in multiple species, direct, standardized comparisons in the clinically relevant canine setting remain limited. In particular, differences reported across studies are difficult to interpret because isolation enzymes, digestion times, plating substrates, and culture conditions often vary between OB and OM protocols, and donor-matched comparisons are uncommon. Here, we addressed this gap by isolating OB- and OM-derived primary cultures from the same donors using harmonized tissue handling, identical enzymatic dissociation and culture conditions, and quantitative readouts. We tested the hypotheses that OB-derived cultures contain a higher fraction of OECs and exhibit higher proliferative activity than OM-derived cultures, while OM-derived cultures retain greater cellular heterogeneity, including epithelial components. Our primary endpoints were (i) culture composition quantified by immunocytochemistry (p75^NTR^ for OECs and fibronectin for fibroblast-like cells, with epithelial morphology quantified separately in OM) and (ii) the Ki-67 labeling index as a measure of cell-cycle activity.

## 2. Materials and Methods

### 2.1. Preparation of Culture Vessel Surfaces

In this study, multiwell test plates (TPP Techno Plastic Products AG, Trasadingen, Switzerland) and chamber slides (Nunc Lab-Tek II Chamber Slide System; Thermo Fisher Scientific, Waltham, MA, USA) were used. The wells of these vessels were coated with either fibronectin or poly-D-lysine. To prepare the fibronectin coating, a solution (Biological Industries, Beit-Haemek, Israel) was diluted at a 1:100 ratio in DPBS (Gibco, Thermo Fisher Scientific). Poly-D-lysine (Sigma-Aldrich, St. Louis, MO, USA) was dissolved in DPBS to reach a final concentration of 50 μg/mL. These solutions were applied to the culture surfaces, ensuring complete coverage, and incubated for 30 min at RT. After incubation, any excess solution was removed, and the wells were washed three times with Hanks’ balanced salt solution (HBSS) (Lonza, Basel, Switzerland), with each wash lasting five minutes. The plates were then air-dried and sterilized using UV radiation for 30 min. The surface preparation of the culture vessels was performed on the same day that cell culture was initiated.

### 2.2. Tissue Collection

Olfactory bulbs and olfactory mucosa were obtained from client-owned dogs that were veterinary patients. Due to the severity of their medical conditions, the animals were humanely euthanized by a licensed veterinarian in accordance with AVMA guidelines [1]. Immediately post-mortem, the cranial cavity was opened. In the frontonasal region, an orthopedic saw was used to excise a fragment of bone, which allowed direct access to the olfactory bulbs. Both olfactory bulbs were carefully removed; from each animal, one bulb was used for cell culture isolation, while the contralateral bulb was processed for histological evaluation.

Subsequently, after removal of the ethmoid bone, the olfactory mucosa was exposed and harvested from the nasal cavity of the same animals. The mucosa from each dog was divided into two portions: one processed for histological examination, and the other subjected to cell isolation.

Tissues were collected immediately after euthanasia without unnecessary delay and transferred directly into sterile transport medium supplemented with Penicillin–Streptomycin. Samples were stored overnight at 4 °C, and all subsequent steps were performed under sterile conditions.

### 2.3. Cell Culture Isolation from Olfactory Bulb

**Figure 1:**
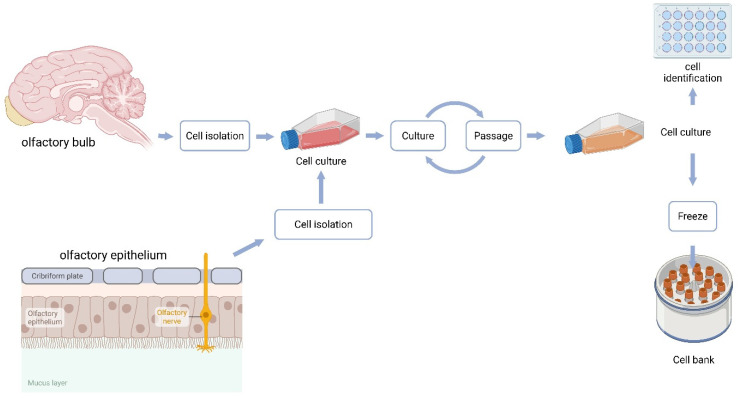
Schematic representation of primary cell culture preparation.

The isolation procedure from the olfactory bulb was performed as follows (Figure 1): The freshly excised bulb designated for cell culture was placed in a Petri dish containing PBS (Lonza, Basel, Switzerland) supplemented with Penicillin–Streptomycin (Gibco, Thermo Fisher Scientific; 100 U/mL penicillin and 100 µg/mL streptomycin, i.e., 1% *v*/*v* from a 100× stock). Under a dissecting microscope (Motic SMZ-168, Motic, Richmond, BC, Canada), blood vessels and meningeal tissues were removed. Tissue mass was determined by weighing the Petri dish before and after sample placement. The bulb tissue was finely minced with a scalpel and transferred into a test tube containing TrypLE (Gibco, Thermo Fisher Scientific; 1×, ready-to-use/undiluted). Enzymatic digestion was carried out at 37 °C in a 5% CO_2_ incubator for 15 min. After digestion, the tissue was gently triturated with a 1 mL pipette and filtered through a cell strainer (Corning Inc., Corning, NY, USA) to obtain a single-cell suspension. The strainer was rinsed with DMEM/F-12 medium (Gibco, Thermo Fisher Scientific) supplemented with 10% fetal bovine serum (FBS; VWR International, Radnor, PA, USA) and Penicillin–Streptomycin (100 U/mL and 100 µg/mL, respectively).

A 25 μL aliquot was taken for viability assessment using a Countstar automated cell counter (ALIT Life Science, Shanghai, China). The remaining suspension was centrifuged at 300× *g* for 5 min, and the pellet was resuspended and plated at a density of 320,000 cells/well (surface area 1.864 cm^2^) in DMEM/F-12 containing 10% FBS. Cells were seeded in both standard culture plates and chamber slide wells (control cultures). Cell confluency was monitored after 48 h, and the medium was refreshed on day 5 post-isolation. Photomicrographs were periodically taken to document cell growth and morphology.

### 2.4. Cell Culture Isolation from Olfactory Mucosa

The mucosal tissue designated for cell culture isolation was processed according to the protocol described by Minkelyte et al. [2], with identical enzymatic digestion, filtration, and culture conditions as used for olfactory bulb–derived cells in this study. Olfactory mucosa (OM) samples designated for cell isolation were rinsed in PBS supplemented with Penicillin–Streptomycin (100 U/mL and 100 µg/mL, respectively), trimmed to remove gross connective tissue, and minced into ~1 mm fragments using sterile scalpels. OM was enzymatically dissociated using the same reagent and incubation conditions as OB (TrypLE™, 1×, ready-to-use/undiluted, 37 °C, 15 min, 5% CO_2_). Following digestion, tissue was gently triturated 10–12 times with a 1 mL pipette to obtain a single-cell suspension, filtered through a 70 µm cell strainer, and the strainer was rinsed with DMEM/F-12 supplemented with 10% FBS and Penicillin–Streptomycin (100 U/mL and 100 µg/mL, respectively). Cells were pelleted (300× *g*, 5 min), resuspended in complete medium, counted, and plated at the same density as OB-derived cultures.

### 2.5. Histological Evaluation

The material for examination was collected post mortem from the brains and nasal cavities. The olfactory bulbs and olfactory regions were dissected for histological evaluation. The material was fixed for 72 h in a 4% buffered formaldehyde solution (pH 7.2–7.4). The tissues were rinsed in running tap water for 24 h, after which the sections were dehydrated by placing them in ethanol solutions of increasing concentration and in xylene. After impregnation with paraffin, they were embedded in blocks. The tissues were cut into 7 μm thick sections and stained classically with haematoxylin and eosin (HE), Alcian blue-PAS and toluidine blue stainings. All reagents were purchased from Merck KGaA (Darmstadt, Germany). Histological preparations were evaluated using a Nikon Eclipse 80i microscope (Nikon, Tokyo, Japan) and a Jenoptik Gryphax^®^ Kapella camera with the Gryphax^®^ software version: 2.2.0 (JENOPTIK Optical Systems GmbH, Jena, Germany).

### 2.6. Viability Assessment

Cell viability was assessed by the trypan blue exclusion method (0.1% trypan blue). A defined volume of 10 µL cell suspension was mixed with trypan blue at (1:1), incubated for 2 min at room temperature, and viable/total cells were counted in a Bürker chamber. For each sample, counts were performed in 4 fields/chamber squares and averaged. Viability was reported as (% viable cells) and total viable yield was calculated as (viable concentration × suspension volume).

### 2.7. Immunofluorescence Procedure

Cells cultured on chamber slides were fixed with 4% paraformaldehyde (PFA; Biotium, Fremont, CA, USA) on the day of collagen gel preparation. After removing the culture medium, PFA was applied for 20 min at room temperature and then discarded. The cultures were washed three times for five minutes with PBST (0.1% Tween 20 in PBS). Cell membrane permeabilization was performed using 0.1% Triton X-100 in PBST for 10 min at room temperature. To block nonspecific antibody binding, the cultures were incubated with 1% bovine serum albumin (BSA) in PBST for 30 min.

Next, the cells were treated with primary antibodies—anti-fibronectin (1:200; Abcam, Cambridge, UK) and anti-p75 NGF receptor (1:100; Sigma-Aldrich)—diluted in 1% BSA in PBST and incubated overnight at 4 °C. After thorough washing with PBST, the cultures were further incubated with species-specific fluorescent secondary antibodies: Anti-Mouse Alexa Fluor 555 (Abcam, cat. no. ab150106) and Anti-Rabbit Alexa Fluor 488 (Abcam, cat. no. ab150077), both diluted 1:500 in PBST. Following final rinses in PBST, the samples were mounted using VECTASHIELD Antifade Mounting Medium (Vector Laboratories, Burlingame, CA, USA) and coverslipped to ensure long-term preservation of stained specimens.

### 2.8. Ki-67 Proliferation Assay

Cells derived from OM and OB were analyzed for Ki-67 expression using the Muse^®^ Ki-67 Proliferation Kit (Merck Millipore, Burlington, MA, USA) according to the manufacturer’s instructions. Adherent cells were detached with TrypLE™ Express (37 °C, 3–5 min), neutralized with complete medium, pelleted (300× *g*, 5 min), and resuspended in PBS. For each condition, 1 × 10^5^ cells were fixed (15 min, room temperature), permeabilized (20 min, protected from light), stained with Ki-67-PE (30 min, room temperature, in the dark), washed, and resuspended in 200 µL Assay Buffer. Samples were acquired immediately on a Muse^®^ Cell Analyzer (Burlington, MA, USA) (≥10,000 events/sample) and analyzed in Muse^®^ Analysis Software v1.8. The workflow comprised debris exclusion by scatter, doublet exclusion using Muse^®^ event processing, and Ki-67(+) thresholding based on unstained and isotype controls and a serum-starved negative control. As Ki-67 was measured in a single PE channel, fluorescence compensation was not applied. The percentage of Ki-67-positive cells was reported within the gated singlet population; a proliferating positive control was included in each run.

### 2.9. Statistical Analysis

All assays (ICC, viability, Ki-67 and morphology) were performed using cells derived from *n* = 10 independent animals (biological replicates). For each assay and condition, measurements were performed in technical triplicates (three independent technical repeats), and the mean of technical replicates was used for statistical comparisons.

Statistical analyses were performed in Tibco Statistica version 13.3 (StatSoft, Kraków, Poland). Normality was assessed using the Shapiro–Wilk test, and homogeneity of variances was evaluated using Levene’s test. For two-group comparisons (OB vs. OM), Welch’s *t*-test was applied when variances were unequal; otherwise, Student’s *t*-test was used. A two-sided *p* < 0.05 was considered significant. Biological replicates represent individual dogs; where technical replicates were present, they were averaged per donor prior to inferential testing.

## 3. Results

### 3.1. Patient Population Characteristics

The study included 10 dogs aged between 4 and 13 years (mean: 10.2 years; median: 10 years) (Table 1).

Mixed-breed dogs (Mix) predominated in the group (6/10), while the remaining breeds were Jack Russell Terrier (JRT), miniature dachshund, English bulldog, and golden retriever (one individual each).

The main reasons for consultation included neoplastic diseases (5 cases), internal medicine disorders (chronic kidney disease, heart disease, multiple organ failure), and mechanical trauma (1 case).

### 3.2. Detailed Histological Characterization of the Olfactory Bulb and Olfactory Mucosa in the Dog

To place the cell-culture findings in tissue context, we first examined the histological architecture of the canine olfactory system using H&E, Alcian Blue–PAS, and toluidine blue stains. This combination captured the overall cyto-architecture, mucous/glycoprotein components, and stromal/nerve bundle organization. Representative fields for the olfactory bulb (OB) and olfactory mucosa (OM) provide the interpretive backdrop for the ICC and Ki-67 results that follow.

**Figure 2:**
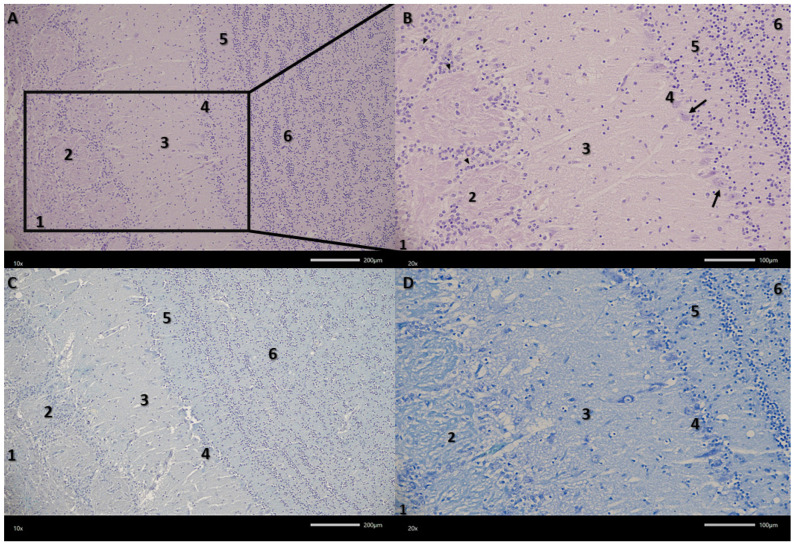
Microphotograph of representative section through the olfactory bulb. (**A**,**B**) Haematoxylin and eosin (HE) staining, (**C**) Alcian blue-PAS staining, (**D**) toluidine blue staining. The six layers of the olfactory bulb: 1—olfactory nerve layer; 2—glomerular layer; 3—external plexiform layer; 4—mitral cell layer; 5—internal plexiform layer; 6—granular cell layer; arrowhead—periglomerular cell; arrow—mitral cell. Magnifications (**A**,**C**) 100×, (**B**,**D**) 200×. Scale bars (**A**,**C**) 200 μm, (**B**,**D**) 100 μm.

The examined olfactory bulbs present a multilayered structure (Figure 2). The first, outermost layer is the olfactory nerve layer with clearly visible olfactory nerve axons covering the surface of the olfactory bulb, organized in intersecting bundles. The next layer in the examined olfactory bulbs is the glomerular layer, containing numerous spherical synaptic glomeruli surrounded by juxtaglomerular cells, mainly periglomerular cells. The third layer in the olfactory bulbs is the external plexiform layer. It is the second widest layer. Its entire surface is covered with clearly visible blood vessels surrounded by pericytes and scattered tufted cells. The next layer in the examined olfactory bulbs is the mitral cell layer, consisting of a single row of mitral cell perikarya and a small number of granule cells. Mitral cells are pyramidal in shape, with a clearly visible cell body and a nucleus with a nucleolus. The last two layers are the internal plexiform layer and the granular cell layer. The granular cell layer is the widest layer, consisting of fairly tightly packed clusters of round granular cells. There are also a large number of sparsely visible blood vessels here.

**Figure 3:**
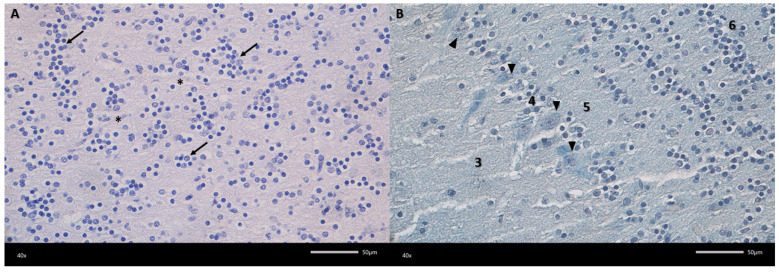
Representative microphotograph of the granular cell layer (**A**) and mitral cell layer (**B**). (**A**) Haematoxylin and eosin (HE) staining, (**B**) Alcian blue-PAS staining. 3—external plexiform layer; 4—mitral cell layer; 5—internal plexiform layer; 6—granular cell layer; arrow—aggregates of granular cells; arrowheads—mitral cell; asterisks—blood vessel. Magnification 400×. Scale bars 50 μm.

Examined olfactory membranes are composed of epithelium and the lamina propria of the mucous membrane (Figure 3). Three types of cells can be distinguished in the epithelium, best visible in Alcian blue-PAS staining. These are olfactory receptor cells, occupying the central part of the epithelium, with an oval nucleus and a visible small cytoplasmic rim. Another type are supporting cells, found at various levels of the epithelium, more often in the upper and lower layers of the epithelium. They have a round nucleus and their cytoplasm is less visible. The cells lying in a single row at the border between the epithelium and the lamina propria are basal cells. In the basal part of the epithelium, pigment granules are visible organized in clusters.

**Figure 4:**
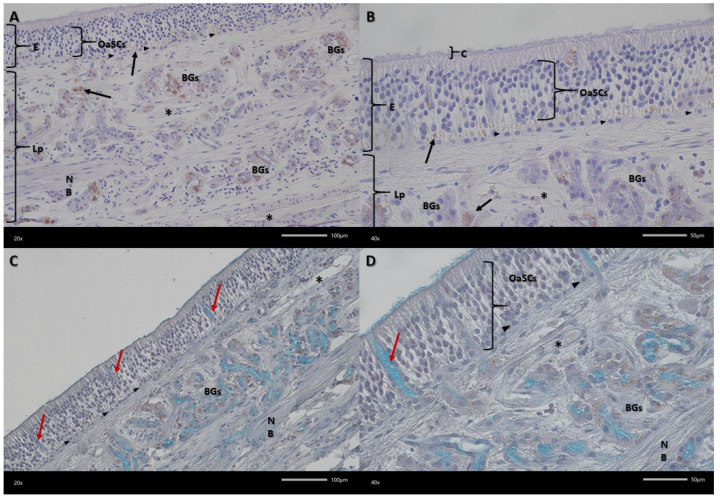
Representative micrograph showing the olfactory mucosa. (**A**,**B**) Haematoxylin and eosin (HE) staining, (**C**,**D**) Alcian blue-PAS staining. OaSCs—olfactory and supporting cells; E—epithelium; Lp—lamina propria of mucosa; C—cilia; BGs—Bowman’s glands; NB—nerve bundle; arrows—pigment grains; red arrows—excretory ducts; arrowhead—basal cells; asterisks—blood vessels. Magnification (**A**,**C**) 200×; (**B**,**D**) 400×. Scale bar (**A**,**C**) 100 μm; (**B**,**D**) 50 μm.

The lamina propria of the mucous membrane is a layer composed of connective tissue (Figure 4). It contains Bowman’s glands, whose excretory ducts release secretions (acidic mucins) onto the surface of the epithelium. Within the epithelium of these glands in dogs, small, densely arranged pigment granules are visible. Within the lamina propria, there are bundles of olfactory nerves and small blood vessels with pericytes.

Overall, the histology corroborates the expected tissue identities and structural landmarks of canine OB and OM, supporting subsequent comparisons between OB- and OM-derived cultures.

### 3.3. Cell Culture and Morphology

Primary cultures established from the olfactory bulb (OB) and olfactory mucosa (OM) adopted distinct, source-dependent phenotypes that were already apparent at low magnification and became striking under higher power. From the outset, OB-derived cultures tended to organize into interlacing strands of spindle/bipolar cells with long, tapering processes suggestive of olfactory ensheathing cell (OEC) morphology. In contrast, OM-derived cultures were more heterogeneous, combining a cobblestone-like epithelial sheet with interspersed elongated fibroblast-like cells, yielding a mosaic growth pattern rather than aligned bundles.

**Figure 5:**
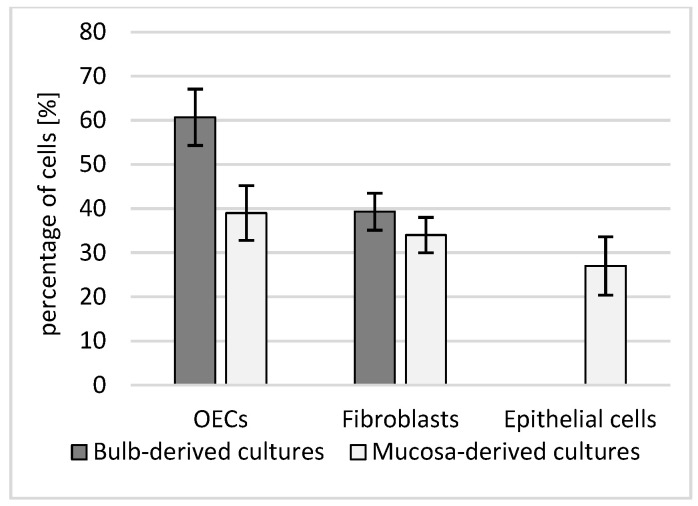
Cellular composition of primary cultures derived from the olfactory bulb (OB) and olfactory mucosa (OM). Mean ± SD percentages of olfactory ensheathing cells (OECs), fibroblasts, and (in OM) epithelial cells quantified on chamber slides. OB cultures contained a higher OEC fraction than OM cultures (Welch’s *t*-test, *p* = 0.014). Values represent biological replicates (dogs); technical duplicates were averaged per donor.

These qualitative impressions were borne out by quantitative composition analysis on chamber slides (Figure 5).

**Figure 6:**
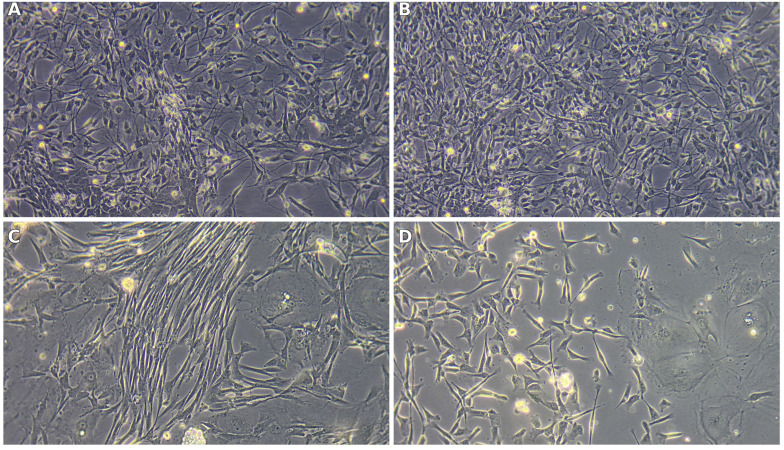
Representative phase-contrast morphology of primary cultures. (**A**,**B**) OB-derived: bipolar/spindle OEC-like cells forming aligned, fascicle-like networks. (**C**,**D**) OM-derived: polygonal epithelial monolayers (cobblestone pattern) interspersed with fibroblast-like cells, reflecting the heterogeneous composition quantified in Figure 4. Scale bars as shown on the micrographs.

OB cultures were enriched in OECs (60.7 ± 6.4%), with a smaller fibroblast component (39.3 ± 4.2%). OM cultures contained OECs (39.0 ± 6.2%), fibroblasts (34.0 ± 4.0%), and a distinct epithelial fraction (27.0 ± 6.6%); the between-source contrast in OEC content reached significance (Welch’s *t*-test, *p* = 0.014; biological replicates = dogs, technical duplicates averaged per donor). Notably, the epithelial compartment quantified for OM in Figure 6 corresponds to the cobblestone epithelial sheet seen on phase-contrast images (Figure 6C,D), whereas OB fields (Figure 6A,B) are dominated by aligned, fascicle-like networks of process-bearing cells consistent with OEC-rich preparations.

**Figure 7:**
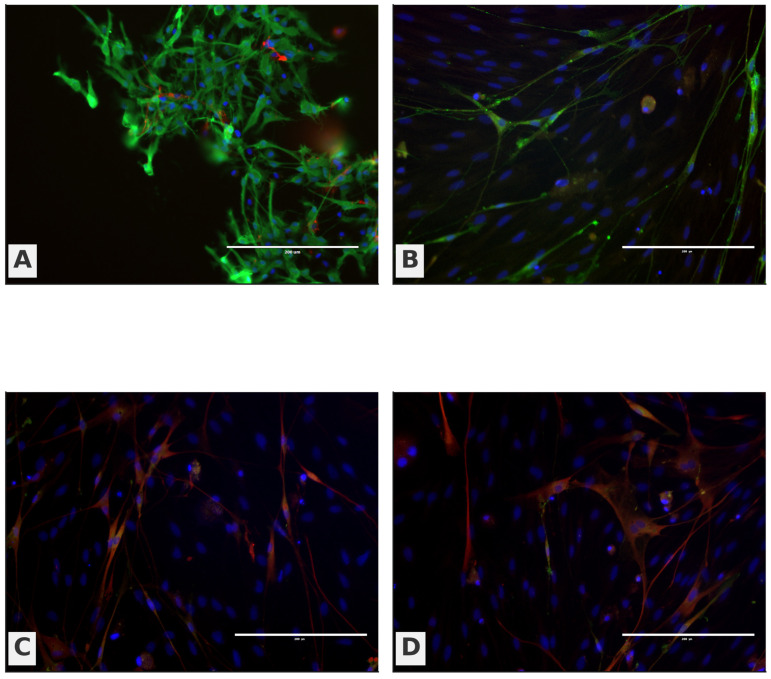
Immunocytochemical characterization of OB- and OM-derived primary cultures. Representative fluorescence images stained for p75^NTR (OEC-associated; green) and fibronectin (fibroblast-associated; red), with DAPI nuclear counterstain (blue). (**A**,**B**) OB-derived cultures show a stronger p75^NTR signal and elongated, process-bearing cells; (**C**,**D**) OM-derived cultures show a weaker p75^NTR signal and a predominance of fibronectin+ fibroblast-like cells surrounding epithelial areas. Scale bars are shown on the micrographs.

Immunocytochemistry (ICC) further reinforced this pattern (Figure 7). OB panels (A–B) display a stronger green OEC/neural signal with elongated neurite-like processes traversing between DAPI-positive nuclei, while OM panels (C–D) show a weaker green signal with a predominance of red fibroblast-like cells arrayed around the epithelial layer. Taken together, Figure 4, Figure 5, Figure 6 and Figure 7 provide a coherent link from cellular composition to phase-contrast morphology and immunophenotype: OB-derived cultures are OEC-biased and process-forming, whereas OM-derived cultures retain an epithelial contingent alongside fibroblasts, yielding a mixed cellular landscape.

### 3.4. Ki-67 Proliferation

**Figure 8:**
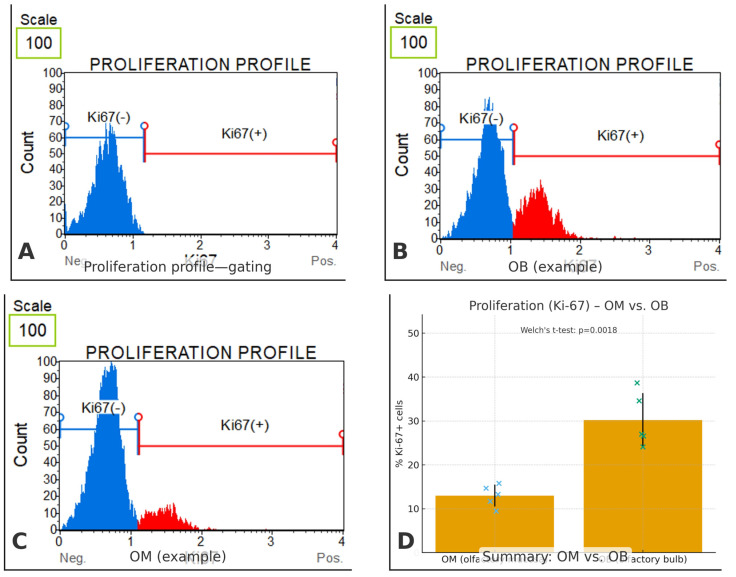
Ki-67-based proliferation in canine olfactory cell cultures. (**A**) Representative Muse^®^ histogram illustrating the gating strategy. Debris and doublets were excluded; the threshold separating Ki-67(–) and Ki-67(+) fractions was set using unstained/isotype and serum-starved negative controls. (**B**) OB (olfactory bulb) example showing a prominent Ki-67(+) peak. (**C**) OM (olfactory mucosa) example with a small Ki-67(+) fraction. (**D**) Summary comparison of % Ki-67(+) cells (mean ± SD; blue crosses = individual OM samples, green crosses = individual OB samples). OM: 13.0% ± 2.5, *n* = 5; OB: 30.2% ± 6.2, *n* = 5. Statistics: Welch’s *t*-test *p* = 0.0018. For each sample, ≥10,000 events were acquired on a Muse^®^ Cell Analyzer and analyzed in Muse^®^ Analysis Software v1.8. Error bars indicate SD. Significance threshold was set at *p* < 0.05.

To gauge proliferative capacity of the canine olfactory cell populations, we quantified the fraction of Ki-67–positive cells in cultures derived from olfactory mucosa (OM) and olfactory bulb (OB) (Figure 8) a 95% CI of 9.67 to 24.73. For completeness, group-wise 95% CIs for the means were 22.55–37.85% (OB) and 9.91–16.09% (OM). The effect size was very large (Hedges’ g = 3.30), indicating a substantial shift toward proliferation in OB-derived cells relative to OM-derived cells. Each measurement was based on ≥10,000 acquired events with debris/doublet exclusion, and values are reported as mean ± SD.

## 4. Discussion

The present study demonstrated that olfactory ensheathing cells (OECs) can be successfully isolated from both the olfactory bulb and the olfactory mucosa of dogs, yet the proportion of OECs within the resulting primary cultures differed significantly between sources. Bulb-derived preparations contained a higher average percentage of OECs (60.7 ± 6.4%), whereas mucosa-derived cultures yielded fewer OECs (39.0 ± 6.2%) and consistently included epithelial cells in addition to fibroblasts. This difference was statistically significant in parametric analysis and indicates that the olfactory bulb provides a richer cellular source of OECs compared to the mucosa. Consistent with this source-dependent contrast, OB-derived cultures displayed a higher Ki-67–positive fraction than OM-derived cultures (30.2 ± 6.2% vs. 13.0 ± 2.5%; Welch’s *t*-test *p* = 0.0018), indicating a larger proportion of actively cycling cells. This proliferative advantage may contribute to faster in vitro expansion and could, in part, underlie a more favorable reparative profile. However, Ki-67 reports cell-cycle status rather than lineage commitment or the direct capacity to support axonal growth, and thus should be interpreted as evidence of proliferative capacity rather than functional efficacy.

The obtained histological findings were consistent with the typical structure of the canine olfactory bulb and mucosa, confirming the correct identification and isolation of the source tissues. This analysis provides additional validation that the differences in OEC proportions between bulb- and mucosa-derived cultures result from genuine biological properties rather than technical errors. Moreover, the distinct histological architecture of the two sources may partly explain the observed cellular heterogeneity in mucosa-derived cultures.

Our findings are consistent with previous reports that OEC content is highly variable depending on the tissue of origin and the isolation method used. Minkelytė et al. (2021) [2] demonstrated that mucosa-derived preparations often contain a substantial admixture of fibroblasts and epithelial cells, which limits the proportion of OECs available for transplantation. This aligns with our observation that mucosal cultures were heterogeneous and yielded fewer OECs compared to bulb-derived isolates.

From a translational perspective, OEC source is commonly a balance between feasibility and culture composition. In the randomized controlled canine trial by Granger et al. (2012), autologous mucosa-derived olfactory cell transplants in chronic spinal cord injury improved fore–hindlimb coordination but did not restore long-tract functions such as voluntary movement or bladder control [3]. Human reports, including Li et al. (1997) and Tabakow et al. (2014), further illustrate both the promise and limitations of OEC-based interventions in complex clinical settings [4,5]. Together, these studies support the view that OM-derived preparations are clinically feasible but may require optimization of enrichment and standardization to reduce heterogeneity and improve reproducibility of the cellular product.

In light of our findings, strategies to enhance the OEC fraction within mucosa-derived isolates deserve particular attention. Approaches such as refined dissociation and plating conditions, selective culture methods, and enrichment workflows (e.g., immunoselection) may help increase the proportion of OECs and reduce epithelial/fibroblast contamination. Future work should directly link composition metrics (p75^NTR^+ fraction, epithelial component, and Ki-67 labeling index) to functional readouts relevant to repair using standardized assays and, ultimately, in vivo validation. Limitations. This study has several limitations. First, the sample size was modest, which reduces statistical power and may limit generalizability across breeds, ages, and sexes. Second, our analyses were primarily descriptive and marker-based; we did not perform functional assays to directly test key OEC properties relevant to regeneration (e.g., neurite outgrowth support, migration, myelination, or pro-regenerative paracrine activity). Third, we did not conduct longitudinal analyses across extended culture time or multiple passages, and therefore cannot conclude how stable the observed OB–OM differences are during expansion. Finally, we did not include in vivo validation, so the translational relevance of the observed in vitro differences remains to be established in appropriate injury models. Accordingly, our findings should be interpreted as a standardized baseline comparison of OB- and OM-derived primary canine cultures that motivates future functional and in vivo work.

## 5. Conclusions

We established primary canine olfactory cell cultures from the olfactory bulb (OB) and olfactory mucosa (OM) under harmonized isolation and culture conditions and demonstrate that the tissue source strongly shapes culture composition and growth. OB-derived cultures contained a higher fraction of p75^NTR+ OECs and exhibited a higher Ki-67 labeling index in vitro, whereas OM-derived cultures were more heterogeneous and included a consistent epithelial component alongside fibroblast-like cells. These results provide a reproducible baseline for selecting and optimizing canine OEC sources, particularly supporting efforts to enrich OECs from clinically accessible OM tissue. Important limitations include the absence of functional assays (e.g., neurite outgrowth support, migration, and myelination), a limited sample size for Ki-67, and a lack of longitudinal passaging and in vivo validation. Future studies should quantify yield, standardize enrichment strategies, and directly test functional regenerative outcomes.

## Figures and Tables

**Table 1:** Characteristics of the study population.

Parameter	Value
Number of patients	10
Age mean (range) [median]	10.2 (4–13) [10] years
Bread–mix (other)	6 (4)
Main reasons for consultation	Neoplasia, CKD, heart disease, trauma

## Data Availability

Administrative data are available upon contacting the first author.
